# Hypoxia preconditioning attenuates lung injury after thoracoscopic lobectomy in patients with lung cancer: a prospective randomized controlled trial

**DOI:** 10.1186/s12871-019-0854-z

**Published:** 2019-11-11

**Authors:** Wenjing Zhang, Mo Chen, Hongbin Li, Jia Yuan, Jingjing Li, Feixiang Wu, Yan Zhang

**Affiliations:** 1 Department of Anesthesiology, Zhoushan Hospital, No.739 Dingshen Street, Zhoushan, Zhejiang China; 2 Department of Anesthesiology, Suzhou Municipal Hospital (North District), Nanjing Medical University Affiliated Suzhou Hospital, No.242 Guangji Road, Suzhou, Jiangsu China; 3 Department of Anesthesiology, Shanghai Eastern Hepatobiliary Surgery Hospital, Second Military Medical University, No.225 Changhai Road, Shanghai, China

**Keywords:** Hypoxic preconditioning, One-lung ventilation, PaO_2_/FiO_2_ ratio, Pulmonary complications, Thoracoscopic lobectomy, Non-small cell lung cancer

## Abstract

**Background:**

Hypoxic preconditioning (HPC) may protect multiple organs from various injuries. We hypothesized that HPC would reduce lung injury in patients undergoing thoracoscopic lobectomy.

**Methods:**

In a prospective randomized controlled trial, 70 patients undergoing elective thoracoscopic lobectomy were randomly allocated to the HPC group or the control group. Three cycles of 5-min hypoxia and 3-min ventilation applied to the nondependent lung served as the HPC intervention. The primary outcome was the PaO_2_/FiO_2_ ratio. Secondary outcomes included postoperative pulmonary complications, pulmonary function, and duration of hospital stay.

**Results:**

HPC significantly increased the PaO_2_/FiO_2_ ratio compared with the control at 30 min after one-lung ventilation and 7 days after operation. Compared with the control, it also significantly improved postoperative pulmonary function and markedly reduced the postoperative hospital stay duration. No significant differences between groups were observed in the incidence of pulmonary complications or overall postoperative morbidity.

**Conclusions:**

HPC improves postoperative oxygenation, enhances the recovery of pulmonary function, and reduces the duration of hospital stay in patients undergoing thoracoscopic lobectomy.

**Trial registration:**

This study was registered in the Chinese Clinical Trial Registry (ChiCTR-IPR-17011249) on April 27, 2017.

## Background

Surgical resections of lobar and mediastinal lymphadenectomy remain the standard for treatment of early stage non-small cell lung cancer (NSCLC). One-lung ventilation (OLV) techniques are required to facilitate the surgical procedure in patients undergoing lung cancer surgery. However, this technique is associated with hypoxia and ischemia in the nondependent lung tissue [1–3]. Moreover, the nondependent lung is reventilated and reoxygenated when the surgical procedure is completed, which triggers a well-characterized hypoxia-reoxygenation injury similar to ischemia-reperfusion injury [4]. Lung injury is associated with significantly prolonged hospital stay and increased rates of postoperative morbidity and mortality. Therefore, prevention and mitigation of OLV-related lung injury would be expected to improve outcomes in patients undergoing lung surgery [5].

Hypoxic preconditioning (HPC) or ischemic preconditioning (IPC) can improve the tolerance of cells, tissues, entire organs, and even the organism itself to subsequent severe hypoxia or ischemia [6–8]. The phenomenon of preconditioning has been observed in various studies [9, 10]. To date, it has been clear that HPC serves as a protective mechanism for multiple organs against various kinds of injuries [6, 11–13]. For example, HPC induced neuroprotection against transient global cerebral ischemia in adult rats [10]. Furthermore, a recent clinical trial found that a 10-min HPC could protect the kidneys from ischemic injury and postsurgical dysfunction in patients undergoing coronary artery bypass surgery [14].

In our study, we hypothesized that HPC performed before surgery would improve oxygenation, reduce postoperative pulmonary complications and enhance recovery after thoracoscopic lobectomy in patients with lung cancer.

## Methods

### Ethics approval and consent to participate

This study was approved by the Institutional Ethics Committee of Zhoushan Hospital (Ref. 2017–008) and was registered in the Chinese Clinical Trial Registry (ChiCTR-IPR-17011249) on April 27, 2017. Our study adhered to CONSORT guidelines. Written consents to participate were obtained from all participants after enrollment.

### Clinical trial course

From April to August 2017, we screened all patients scheduled for thoracoscopic lobectomy for clinical stage I or II NSCLC. The inclusion criteria for participants of this study were aged 18 to 75 years, with an American Society of Anesthesiologists physical status of I or II. The exclusion criteria of this study were severe pulmonary dysfunction or difficulty intubation of double-lumen tube (DLT).

Patients were randomly allocated to two groups (1:1) based on computer-generated random numbers. Allocation details were sealed in sequentially numbered opaque envelopes. All patients, surgeons and research staff performing follow-up were fully blinded to group allocation. The anesthetists were not blinded and were not involved in study follow-up.

To avoid interference with the trial intervention, we used a standardized perioperative management protocol (including thoracic anesthesia, fluid management, surgical approach, and postoperative care). No patients received premedication. All patients were fasted 6 h to solids and 2 h to clear liquids. In the operating room, a radial arterial line was inserted for measuring the arterial pressure and sampling the arterial blood gas. For induction, patients received midazolam (0.05 mg/kg), sufentanil (5 μg/kg), propofol (1.5 mg/kg), and cisatracurium (0.2 mg/kg). After induction, an appropriate size of DLT was intubated, and the correct position of DLT was confirmed by fiberoptic bronchoscope in both supine and lateral positions. The fraction of inspired oxygen (FiO_2_) was initially set at 60%, and in cases of saturation of pulse oxygenation (SpO_2_) less than 92%, FiO_2_ was increased to 100%. Anesthesia was maintained with 1.0 to 2.5% sevoflurane, remifentanil, and cisatracurium. The protocol for ventilation was as follows: tidal volume of 8 ml/kg per ideal body weight during two-lung ventilation (TLV) and 6 ml/kg per ideal body weight during OLV, and respiratory rate of 12/min without positive end-expiratory pressure. All patients received 4 mL/kg/h Ringer’s lactate solution throughout the intraoperative period. In the postoperative period, maintenance fluids (2 mL/kg/h) were continued until patients were able to tolerate adequate oral intake. All operations were performed by an experienced thoracic surgical team specialized in video-assisted thoracoscopic surgery. At the end of the surgery, the tracheal tube was removed, and patients were transferred to the postanesthesia care unit. Ultrasound-guided unilateral thoracic paravertebral nerve block (T_5–6_ and T_6–7_) was used by injecting 10 mL of ropivacaine 0.25% for postoperative analgesia. All patients received additional analgesic treatment with paracetamol 1 g 4 times daily.

### Intervention

In the HPC group (*n* = 38), HPC was applied after confirmation of the DLT position in the correct positions and before incision. HPC was applied on the nondependent lung by intermittent ventilation. Three cycles were performed, each consisting of 5-min of no ventilation and opening to atmosphere followed by 3-min of ventilation. The protocol for ventilation was as follows: tidal volume of 6 ml/kg per ideal body weight, respiratory rate of 12/min, FiO_2_ of 60%, sevoflurane of 1.5% and intermittent two-lung ventilation when SpO_2_ < 92%. The non-operated lung received continuous ventilation. In the control group (*n* = 38), patients received TLV after confirmation of the DLT position and before incision.

### Outcome

The primary outcome was lung oxygenation expressed by the PaO_2_/FiO_2_ ratio. Secondary outcomes included the following: intraoperative adverse events, pulmonary function, postoperative pulmonary complications, and duration of hospital stay.

Arterial blood gases were evaluated at 30 min after OLV and at 7 day after surgery. The occurrence of intraoperative adverse events, such as tachycardia, bradycardia, hypotension, hypertension, and oxygen desaturation were recorded by the clinical anesthesiologists. Hypotension and bradycardia were defined as a decrease from baseline value by 20%. Hypertension and tachycardia were defined as an increase from baseline value by 20%. Oxygen desaturation was defined as a SpO_2_ value of < 92%. We used a portable spirometer (Spirobank, GTM, Medical International Research, Rome, Italy) to measure the pulmonary function of forced vital capacity (FVC) and forced expiratory volume in 1 s (FEV_1_) before surgery and repeated this measurement at 7 day after surgery. Every pulmonary function test was performed three times with the patient in a semirecumbent position, and the average value was recorded. Postoperative pulmonary complications were defined as pneumonia, atelectasis, pleural effusions, and prolonged air leak > 7 days. The duration of hospital stay was counted from the day of operation to the day of discharge.

### Statistical analysis

Sample size calculation was based on the primary outcome of the PaO_2_/FiO_2_ ratio. A previous study showed that the means ± standard deviation (SD) value of PaO_2_/FiO_2_ during OLV was 192 ± 67 mmHg [15]. Our hypothesis was that the HPC would improve the PaO_2_/FiO_2_ ratio by 25%. Assuming a power of 80% and a level of significance of 0.05, it was estimated that 31 patients would be required for each group. To account for a 10% dropout rate, we included 35 patients in each group.

Statistical analyses were conducted using the SPSS 20.0 statistical software (IBM Corporation, Armonk, NY, USA). Quantitative data were expressed in the means ± SD or medians (interquartile range, IQR) and compared with independent *t*-test or Mann-Whitney *U* test, respectively. Categorical data were expressed as a frequency or percentage and compared with the Fisher’s exact test or the chi-square test. Variables with repeated measures were analyzed using a linear mixed model with a patient indicator as a random effect and group, time, and group-by-time as fixed effects. *P* values less than 0.05 were considered to be statistically significant.

## Results

**Fig. 1:**
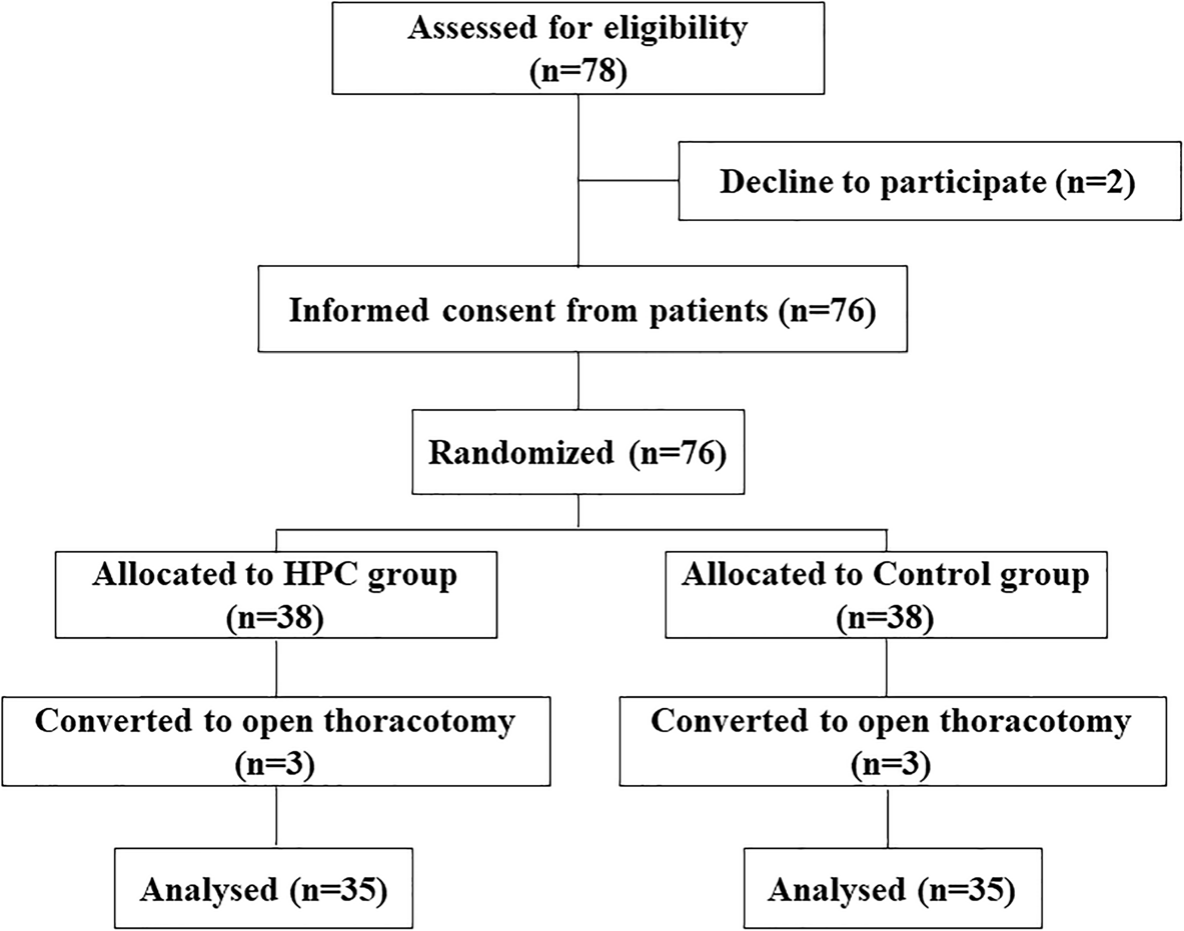
Participant flow of Hypoxic Preconditioning

A flow diagram of this study is shown in Fig. 1. A total of 78 patients were screened for eligibility, and eight patients were excluded from analysis. Patients were randomized equally between the HPC and Control groups.

Patient characteristics, and pulmonary function test results are shown in Table 1. No differences were observed between two groups regarding age, sex, weight, height, comorbidities, and preoperative pulmonary function.

**Table 1:** Patient characteristics

	HPC group (*n* = 35)	Control group (*n* = 35)
Age (y)	63.1 ± 12.7	62.3 ± 13.5
Sex (male/female)	21/14	19/16
Weight (kg)	60.6 ± 10.9	58.7 ± 11.4
Height (cm)	165 ± 9	162 ± 8
Comorbidities		
Hypertension	8	9
CAD	1	2
Diabetes	0	1
Smoking		
Non-smoker	12	10
Ex-smoker	11	12
Current smoker	12	13
% of predicted FVC	92.3 ± 8.8	91.1 ± 9.2
% of predicted FEV_1_	88.9 ± 10.2	86.2 ± 11.6

Values are expressed as the means ± standard deviation or number (n)

*Abbreviations*: *CAD* coronary artery disease, *FVC* forced vital capacity, *FEV*_*1*_ forced expiratory volume in 1 s, *HPC* hypoxic preconditioning

Intraoperative data is summarized in Table 2. The duration of surgery, types of resections, estimated blood loss, and incidence of adverse events were similar between the two groups.

**Table 2:** Intraoperative characteristics

	HPC group (*n* = 35)	Control group (*n* = 35)	*P* value
Left/right lobectomy	18/17	19/16	0.81
Anesthesia time (min)	213 ± 41	220 ± 45	0.50
Operation time (min)	164 ± 39	171 ± 43	0.48
OLV time (min)	145 ± 27	142 ± 31	0.67
FiO_2_ (%)	97.7 ± 9.4	100	0.16
Crystalloid (ml)	812 ± 65	843 ± 72	0.33
Colloid (ml)	215 ± 46	201 ± 52	0.53
Estimated blood loss (ml)	150 (50–200)	160 (50–250)	0.58
Adverse events (%)	14 (40%)	12 (34.3%)	0.62
Tachycardia	3	1	0.61
Bradycardia	2	5	0.43
Hypotension	4	2	0.67
Hypertension	5	3	0.71
Oxygen desaturation	0	1	0.31

Values are expressed as the means ± standard deviation, median (interquartile range), number or percentage of patients

As shown in Table 3, the PaO_2_/FiO_2_ ratio in the HPC group was significantly higher than that in the control group at 30 min after OLV and 7 day after operation. In addition, postoperative values of FVC and FEV_1_ were significantly greater in the HPC group than in the control group.

**Table 3:** Changes in the PaO_2_/FiO_2_ ratio and in pulmonary function

	Time	HPC	Control
PaO_2_/FiO_2_	OLV 30 min	2.01 ± 0.53^*^	1.83 ± 0.64
	7d after surgery	1.95 ± 0.38^*^	1.74 ± 0.32
FVC	Baseline	2.84 ± 0.66	2.72 ± 0.97
	7d after surgery	2.77 ± 0.93^*^	2.54 ± 0.86
FEV_1_	Baseline	2.32 ± 0.50	2.28 ± 0.80
	7d after surgery	2.27 ± 0.38^*^	2.06 ± 0.57

Values are expressed as the means ± standard deviation. ^*^P < 0.05 compared with control group

*Abbreviations*: *FVC* forced vital capacity, *FEV*_*1*_ forced expiratory volume in 1 s, *HPC* hypoxic preconditioning; OLV, one-lung ventilation

Postoperative complications, mortality, and duration of hospital stay are shown in Table 4. No significant differences were observed between groups in the incidence of pulmonary complications or overall postoperative morbidity. HPC was associated with a statistically significant reduction in postoperative hospital stay.

**Table 4:** Postoperative complications, mortality, and duration of hospital stay

	HPC group (*n* = 35)	Control group (*n* = 35)
Pulmonary complications		
Atelectasis	3	1
Pneumonia	3	4
Prolonged air leak	0	0
Pleural effusion	0	1
Others		
Urinary tract infection	1	0
Neurologic complications	0	0
Overall morbidity	7	6
Mortality	0	0
Postoperative hospital stay (d)	8.32 ± 3.77^*^	10.87 ± 6.58

Values are expressed as the means ± standard deviation, number of patients. ^*^P < 0.05 compared with control group

*Abbreviations*: *HPC* hypoxic preconditioning

## Discussion

This randomized controlled trial demonstrated that HPC performed before surgery improves postoperative oxygenation, enhances recovery of pulmonary function, and reduces the duration of hospital stay in patients who underwent thoracoscopic lobectomy. These results support our hypothesis that the application of HPC on the nondependent lung protects lungs from injury. In addition, no differences in intraoperative adverse events were observed with and without HPC.

The HPC intervention used in this study can also be referred to as intermittent ventilation or intermittent hypoxia. A previous study has demonstrated that intermittent hypoxia increased hypoxic pulmonary vasoconstriction of the nondependent lung [16]. Another previous study also demonstrated that intermittent ventilation had a beneficial effect on oxygenation during OLV for thoracic surgery [17]. However, previous studies only assessed the effect of HPC on intraoperative oxygenation.

In the current study, we assessed the protective effect of HPC on lung injury in patients undergoing thoracoscopic lobectomy. The PaO_2_/FiO_2_ ratio was chosen as the primary outcome because it is a useful indicator for detecting impaired gas exchange and oxygenation among patients receiving a wide range of FiO_2_ [18]. The current study demonstrated a positive effect of HPC that not only mitigated the decrease of the PaO_2_/FiO_2_ ratio during OLV but also improved postoperative oxygenation.

Lung injury following thoracic surgery has been recognized as a major cause of postoperative complication, mortality, and delayed discharge [19–21]. The exact mechanism of lung injury is still unclear. Previous studies have found that lung re-expansion provoked severe oxidative stress, which was associated with major adverse effects after lung resection [22–24]. Moreover, HPC performed before OLV was shown to alleviate systematic inflammatory response and oxidative stress in patients with lung cancer [25]. Thus, we postulated that the protective effect of HPC on lung injury was partly due to the attenuation of oxidative stress in this study. Although a trend toward a reduced incidence of postoperative pulmonary complications was observed in patients who received HPC, this did not reach statistical significance. A possible explanation is that the small sample size of this study may be insufficient to detect this difference between the two groups.

Pulmonary function after thoracic surgery reduced in both groups. The current study found that lung function was better preserved in patients who received HPC than in those who did not receive HPC. These results suggest that the use of HPC is beneficial to the recovery of pulmonary function. In a previous study of an animal model, intermittent hypoxia was shown to be an effective approach for improving respiratory function after cervical spinal cord injury [26].

The primary limitation of our preliminary trial was the relatively small size of the study population. Given the intrinsic biases of a small sample, the findings of the present study should be confirmed with further studies.

## Conclusions

In conclusion, our study suggests that HPC may be a promising prevention option for lung injury, especially in patients undergoing lung cancer surgery. HPC performed before OLV improves postoperative oxygenation, enhances recovery of pulmonary function, and reduces the duration of hospital stay.

## Data Availability

The datasets used and/or analysed during the current study are available from the corresponding author on reasonable request.

## Abbreviations

DLT: Double-lumen tube
FEV_1_: Forced expiratory volume in 1 s
FiO_2_: Fraction of inspired oxygen
FVC: Forced vital capacity
HPC: Hypoxic preconditioning
IPC: Ischemic preconditioning
NSCLC: Non-small cell lung cancer
OLV: One-lung ventilation
SpO_2_: Saturation of pulse oxygenation
TLV: Two-lung ventilation
